# Cycloastragenol Attenuates Cisplatin‐Induced Ovarian Toxicity in Rats: Associations With TERT Expression, Apoptotic Balance, and FOXO3a–mTOR‐Related Signaling

**DOI:** 10.1002/jbt.71152

**Published:** 2026-10-25

**Authors:** Gökçe Nur Arık Erol, Atiye Seda Yar, Gülnur Take Kaplanoğlu

**Affiliations:** ^1^ Department of Histology and Embryology Faculty of Medicine Gazi University Ankara Türkiye; ^2^ Department of Medical Biology, Faculty of Medicine Gazi University Ankara Türkiye

**Keywords:** BAX/BCL‐2 ratio, cisplatin, cycloastragenol, FOXO3a, ovarian toxicity, TERT

## Abstract

Cisplatin (CIS)‐induced ovarian toxicity is characterized by follicular depletion, apoptosis, and disruption of intracellular survival signaling. This study investigated whether cycloastragenol (CAG), a telomerase‐associated bioactive compound, attenuates CIS‐induced ovarian injury in rats. Forty female Sprague–Dawley rats were assigned to two vehicle‐control groups and CAG, CIS, or CIS + CAG groups for 15 days. Vehicle controls were pooled after confirming no significant differences. Ovarian morphology and follicle counts were evaluated histologically. Immunohistochemical analyses included PCNA, caspase‐3, total FOXO3a, phospho‐FOXO3a (Ser253), total mTOR, BAX, BCL‐2, and TERT. Serum anti‐Müllerian hormone and telomerase‐related measurements were assessed by ELISA, and relative telomere length was determined by quantitative real‐time PCR. CIS reduced primordial follicle numbers, increased follicular atresia, enhanced BAX expression, reduced BCL‐2 and TERT immunoreactivity, and increased the BAX/BCL‐2 ratio. CAG co‐treatment significantly attenuated the changes in BAX, BCL‐2, the BAX/BCL‐2 ratio, and TERT. Nuclear phospho‐FOXO3a was significantly lower in the CIS + CAG group than in the CIS group, whereas cytoplasmic phospho‐FOXO3a did not differ significantly. Total mTOR and caspase‐3 were numerically reduced by CAG co‐treatment but did not remain significant after multiple‐comparison correction. PCNA, total FOXO3a, and serum AMH showed partial or nonsignificant recovery. Relative telomere length did not differ among groups. CAG partially attenuated CIS‐induced ovarian injury and apoptotic imbalance and was associated with restoration of ovarian TERT immunoreactivity and modulation of phospho‐FOXO3a localization. However, these findings do not establish a causal telomerase–FOXO3a–mTOR mechanism.

## Graphical abstract

Cycloastragenol partially attenuated cisplatin‐induced ovarian toxicity and was associated with partial preservation of follicular morphology, improvement in apoptotic balance, and changes in FOXO3a–mTOR‐related markers and ovarian TERT expression.

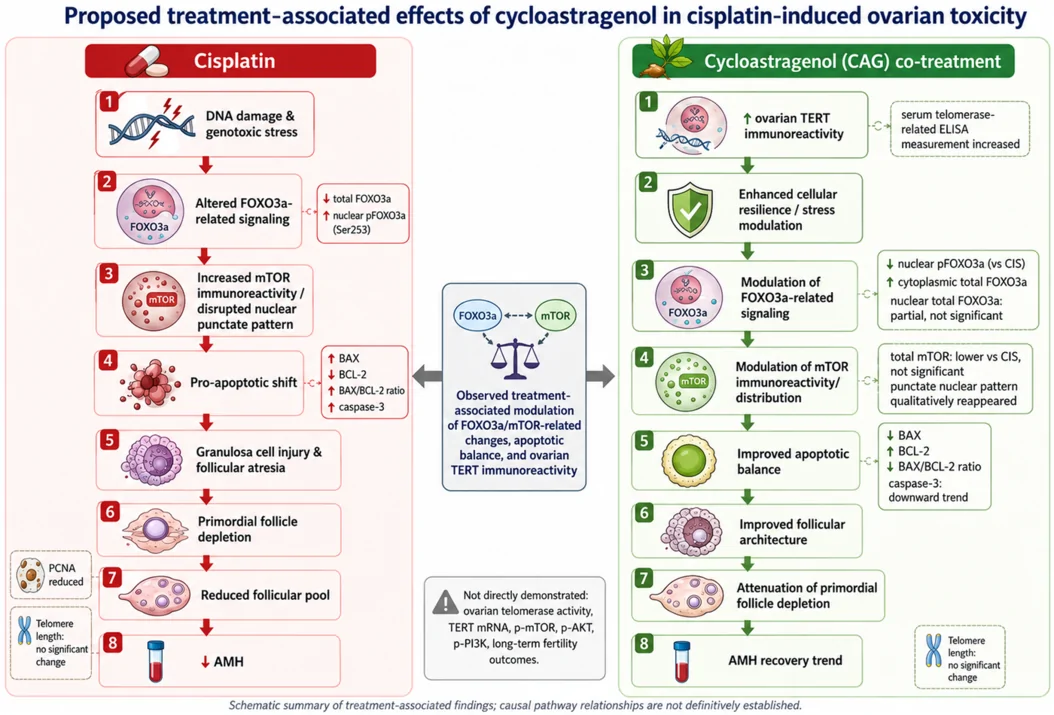

## 1 Introduction

Cisplatin (CIS) is a widely used platinum‐based chemotherapeutic agent whose antitumor activity is primarily mediated through DNA damage and the induction of apoptotic cell death. However, its limited tissue selectivity can cause substantial ovarian toxicity, including granulosa‐cell injury, follicular atresia, depletion of the primordial follicle pool, diminished ovarian reserve, and premature ovarian insufficiency [1, 2]. Because primordial follicles constitute the finite ovarian reserve, their loss during chemotherapy may result in persistent reproductive and endocrine consequences.

The survival and activation of primordial follicles are regulated by interconnected intracellular pathways, particularly the PI3K/AKT/FOXO3a and mTOR signaling networks. Nuclear FOXO3a contributes to the maintenance of primordial follicle quiescence, whereas AKT‐associated FOXO3a phosphorylation and altered subcellular localization are linked to follicular activation [3, 4, 5, 6, 7, 8]. CIS has been reported to disrupt this regulatory balance and promote excessive recruitment of dormant follicles, thereby accelerating depletion of the ovarian reserve [3, 4]. mTOR signaling contributes to primordial follicle activation, oocyte growth, granulosa‐cell function, and follicular survival [9, 10, 11, 12, 13]. Nevertheless, changes in total FOXO3a and total mTOR immunoreactivity alone cannot establish pathway activation. Evaluation of phosphorylation status and subcellular localization may therefore provide additional information regarding treatment‐associated signaling alterations.

Apoptosis represents another major component of CIS‐induced ovarian injury. BAX promotes mitochondrial membrane permeabilization and pro‐apoptotic signaling, whereas BCL‐2 opposes this process and supports cellular survival. Consequently, the balance between BAX and BCL‐2, together with caspase‐3 immunoreactivity, provides an indication of the ovarian apoptotic response [14, 15, 16]. Experimental studies have shown that attenuation of mitochondrial dysfunction and apoptotic signaling may reduce chemotherapy‐associated follicular damage [14, 15, 16, 17].

Telomerase is a ribonucleoprotein complex involved in telomere maintenance and cellular replicative capacity [18]. Its catalytic subunit, telomerase reverse transcriptase (TERT), also participates in telomere‐independent processes, including mitochondrial homeostasis, oxidative‐stress responses, DNA‐damage regulation, and apoptosis [19]. Cycloastragenol (CAG), a triterpenoid compound derived from *Astragalus membranaceus*, has been reported to enhance telomerase‐related activity and TERT‐associated responses in experimental models [20, 21, 22]. It has also shown cytoprotective, anti‐apoptotic, antioxidant, anti‐inflammatory, and anti‐senescent effects [21, 22, 23, 24]. CAG has further been associated with attenuation of age‐related ovarian dysfunction and improvement of telomerase‐associated responses in ovarian tissue [22]. However, its effects on CIS‐induced ovarian toxicity and the relationships among ovarian TERT immunoreactivity, apoptotic balance, and FOXO3a–mTOR‐related signaling remain incompletely understood.

Therefore, the present study investigated whether CAG attenuates CIS‐induced ovarian injury in rats. Ovarian morphology, follicle counts, serum anti‐Müllerian hormone (AMH) levels, serum telomerase‐related measurements, relative telomere length, and immunoreactivity for TERT, BAX, BCL‐2, caspase‐3, PCNA, total FOXO3a, phospho‐FOXO3a (Ser253), and total mTOR were evaluated. The study was designed to identify treatment‐associated changes in ovarian structure, apoptotic balance, TERT immunoreactivity, and FOXO3a–mTOR‐related protein distribution rather than to establish a definitive causal signaling mechanism.

## 2 Materials and Methods

### 2.1 Chemicals and Reagents

CAG (Lot: SMB00372) was purchased from Sigma‐Aldrich (Germany) and stored at 4°C. Prior to administration, CAG was freshly dissolved in 5% dimethyl sulfoxide (DMSO; Merck, Germany). CIS was obtained from MedChemExpress and dissolved in 5% DMSO. The final DMSO concentration did not exceed 5% in any treatment. All other chemicals were of analytical grade.

### 2.2 Animals and Ethics Approval

All experimental procedures were approved by the Institutional Animal Ethics Committee of Gazi University (Approval Code: G.Ü. ET‐22.018) and conducted in accordance with the ARRIVE 2.0 guidelines and the NIH Guide for the Care and Use of Laboratory Animals. Forty healthy female Sprague–Dawley rats (180–200 g, 8–12 weeks old) were housed under controlled conditions (22 ± 3°C, 12 h light/dark cycle, 55 ± 10% humidity) with ad libitum access to food and water. Animals were acclimatized for at least 7 days before the experiments. Randomization was applied for group allocation. Anesthesia was induced by intraperitoneal injection of ketamine (50 mg/kg) and xylazine (10 mg/kg). At the end of the experimental period, animals were deeply anesthetized with a high dose of the same anesthetic agents and euthanized by exsanguination via cardiac puncture.

### 2.3 Experimental Design

Rats were randomly assigned to five groups: oral DMSO (*n* = 5), intraperitoneal DMSO (*n* = 5), CAG (*n* = 10), CIS (*n* = 10), and CAG + CIS (*n* = 10). CAG was administered orally at 7 mg/kg/day, while CIS was administered intraperitoneally at 2 mg/kg/day for 15 consecutive days. The CAG dose of 7 mg/kg/day was selected as an experimental pharmacological dose based on a previous in vivo rat study using oral CAG doses of [3, 14] and 14 mg/kg/day [25]. It does not represent an established ovary‐specific therapeutic dose. As no significant differences were observed between the vehicle control groups (*p* > 0.05), they were pooled for statistical analyses.

### 2.4 Histological Processing and Follicle Quantification

Ovarian tissues were serially sectioned at a thickness of 4.5 µm, and six consecutive sections from one ovary of each animal were selected for histomorphometric analysis. Consecutive sections were evaluated because trimming or introducing wider sampling intervals resulted in loss of small primordial follicles from the examined tissue level. Follicle counting was performed using a systematic evaluation procedure under a ×40 objective. Follicles were classified as primordial, preantral, or atretic based on morphological criteria described by Pedersen and Peters. Preantral follicles were defined as growing follicles surrounded by one or more layers of cuboidal granulosa cells without a visible antral cavity. Follicles exhibiting degenerative changes in the oocyte and/or granulosa cell layers were classified as atretic. Only follicles containing a clearly visible oocyte nucleus were included to reduce the likelihood of counting tangential follicular profiles. For each animal, the mean follicle count obtained from the six evaluated sections was calculated and used as the experimental unit for statistical analysis [26].

### 2.5 Immunohistochemistry

Sections were deparaffinized, rehydrated, and subjected to heat‐induced antigen retrieval in citrate buffer (pH 6.0). Endogenous peroxidase activity was blocked using 3% H_2_O_2_. Sections were incubated overnight at 4°C with primary antibodies against PCNA (Santa Cruz Biotechnology, lot no. J2219; 1:100), total mTOR (Affinity Biosciences, cat. no. AF6308; 1:100), total FOXO3a (Affinity Biosciences, cat. no. AF6020; 1:75), caspase‐3 (Bioss, lot no. AG12061316; 1:200), BCL‐2 (Santa Cruz Biotechnology, cat. no. sc‐7382; 1:50), BAX (Novus Biologicals, cat. no. NB120‐7977; 1:100), TERT (Affinity Biosciences, cat. no. DF7129; 1:100), and phospho‐FOXO3a (Ser253) (Affinity Biosciences, cat. no. AF3020; 1:100).

After incubation with the secondary antibody (Thermo Scientific, lot no. PHLT811) and the streptavidin–peroxidase complex, immunoreactivity was visualized using AEC chromogen (Thermo Scientific, lot no. HA53704). Sections were counterstained with hematoxylin. Routine negative‐control sections were processed in parallel by omitting the primary antibody to assess nonspecific or background staining; these omission controls were not considered independent validation of antibody specificity. Immunohistochemical evaluations were performed independently by two observers blinded to group allocation. Immunoreactivity was evaluated semi‐quantitatively using the *H*‐score method, with nuclear and cytoplasmic staining assessed separately where applicable. PCNA immunoreactivity was evaluated as positive‐cell density.

### 2.6 Serum AMH and Telomerase‐Related ELISA Measurements

Blood samples were collected by intracardiac puncture at the end of the experimental period. Serum was separated by centrifugation and stored at −80°C until analysis. Serum AMH concentrations were measured using a rat MIS/AMH ELISA kit (BT LAB, Shanghai, China; Cat. No. E0456Ra), and serum telomerase‐related concentrations were measured using a rat telomerase ELISA kit (BT LAB, Shanghai, China; Cat. No. E0218Ra), according to the manufacturer's instructions. Absorbance was measured at 450 nm using a microplate reader, and serum concentrations were calculated from the corresponding standard curves.

### 2.7 Quantitative Real‐Time PCR Analysis

Genomic DNA was isolated from ovarian tissue samples using an Exgene Blood SV Maxi kit (GeneAll Biotechnology, Seoul, Republic of Korea; Cat. No. 105‐326) according to the protocol applied in the laboratory. Relative telomere length was assessed using a Rat Telomere Length Quantification qPCR Kit (ELK Biotechnology, Wuhan, China; Cat. No. EQ. 028) according to the manufacturer's instructions.

Quantitative real‐time PCR reactions were performed in duplicate using a QuantStudio 5 Real‐Time PCR System (Applied Biosystems, Thermo Fisher Scientific, Waltham, MA, USA). Telomere amplification was normalized to the reference gene provided with the kit. Relative telomere length was calculated using the comparative $2^{-\Delta \Delta Cq}$ method and expressed relative to the control group.

### 2.8 Statistical Analysis

Statistical analyses were performed using IBM SPSS Statistics, version 25.0 (IBM Corp., Armonk, NY, USA). The animal was considered the experimental unit in all analyses. Before the primary analyses, the oral and intraperitoneal vehicle‐control groups were compared. Because no statistically significant differences were detected between these groups, their data were pooled into a single control group, resulting in four groups for the subsequent analyses.

The distribution of each variable was assessed using the Shapiro–Wilk test. Because the assumptions of normality were not met for the analyzed outcomes, differences among the four groups were evaluated using the Kruskal–Wallis test. When a statistically significant overall group difference was detected, post hoc pairwise comparisons were performed using two‐tailed Mann–Whitney *U* tests. All six possible pairwise comparisons were conducted, and the resulting *p* values were adjusted using the Bonferroni method. Bonferroni‐adjusted *p* < 0.05 were considered statistically significant.

PCNA immunoreactivity was quantified as PCNA‐positive cell density. Immunoreactivity for caspase‐3, total FOXO3a, phospho‐FOXO3a (Ser253), total mTOR, BAX, BCL‐2, and TERT was evaluated using the *H*‐score method. Nuclear and cytoplasmic total FOXO3a and phospho‐FOXO3a (Ser253) immunoreactivity were scored and analyzed separately. The BAX/BCL‐2 ratio was calculated separately for each animal using the paired animal‐level BAX and BCL‐2 *H*‐scores. For each outcome, the mean value obtained from the evaluated sections and microscopic fields was calculated for each animal and used in the statistical analysis, thereby avoiding pseudoreplication.

Follicle counts, PCNA‐positive cell density, serum AMH levels, serum telomerase‐related concentrations, relative telomere length, and immunohistochemical *H*‐scores are presented as median and interquartile range (Q1–Q3). All statistical tests were two‐tailed.

The separate results of the oral and intraperitoneal vehicle‐control groups are provided in Table S1.

### 2.9 Sample Size and Power Analysis

Sample size was calculated using G*Power (version 3.1) based on primordial follicle count as the primary outcome variable. An a priori power analysis was performed for the Kruskal–Wallis test (four groups), assuming a large effect size (*f* = 0.60), *α* = 0.05, and power (1 − β) = 0.80. The minimum required total sample size was estimated as 34 animals. To compensate for potential losses and ensure adequate statistical robustness, 40 rats were included in the study.

## 3 Results

### 3.1 Histopathological Findings (H&E Staining)

In the vehicle control groups, ovarian architecture was preserved, with intact germinal epithelium, organized granulosa cell layers, and well‐defined theca structures (Figure 1A,B). The CAG‐treated group showed morphology comparable to that of the controls, with a predominance of healthy preantral follicles (Figure 1C).

**Figure 1:**
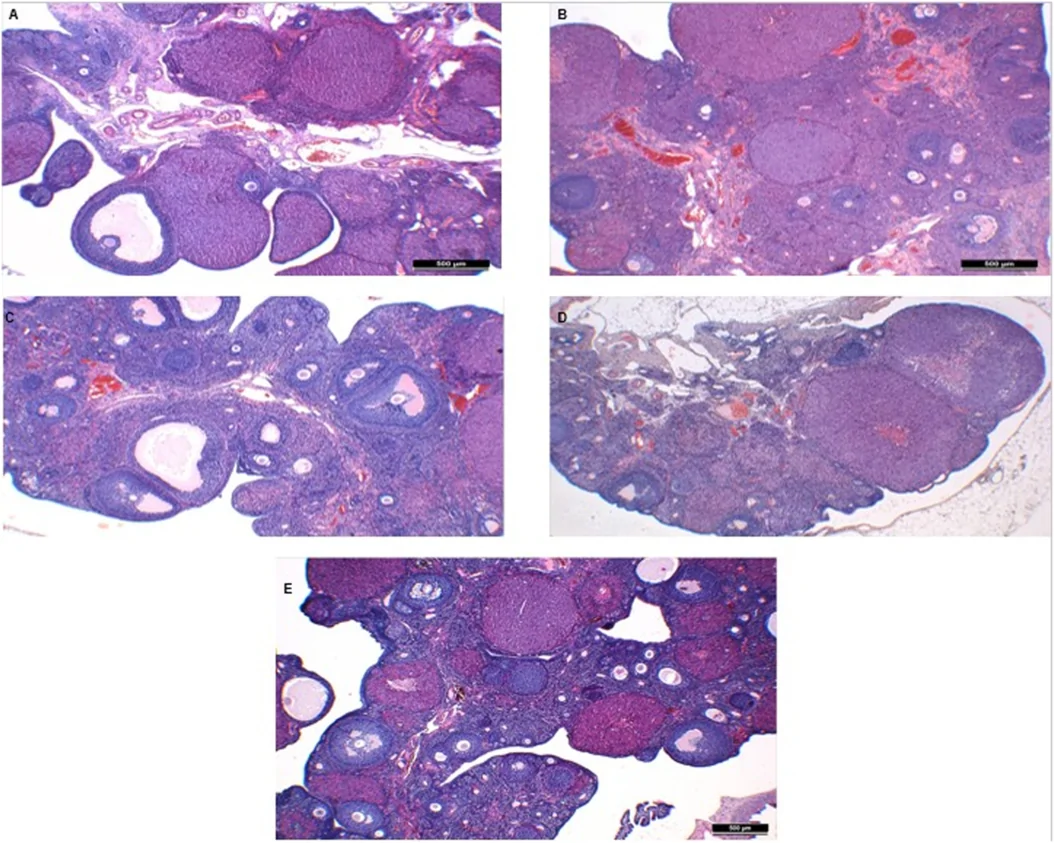
Representative hematoxylin and eosin–stained ovarian sections from the vehicle–gavage control (A), vehicle–intraperitoneal DMSO control (B), cycloastragenol (CAG) (C), cisplatin (CIS) (D), and CIS + CAG (E) groups. Preserved ovarian architecture and follicles at different developmental stages are visible in the control and CAG groups. The CIS group shows reduced follicular density and structural alterations, whereas the CIS + CAG group displays greater follicular preservation than the CIS group. Scale bar = 500 μm.

Quantitative analysis showed that primordial follicle counts in the CAG group were similar to those in the control group (Figure 2A, *p* = 1.000), whereas preantral follicle numbers were significantly increased (Figure 2A, *p* = 0.009). Atretic follicle numbers were significantly lower in the CAG group than in the CIS group (Figure 2A, *p* < 0.001).

**Figure 2:**
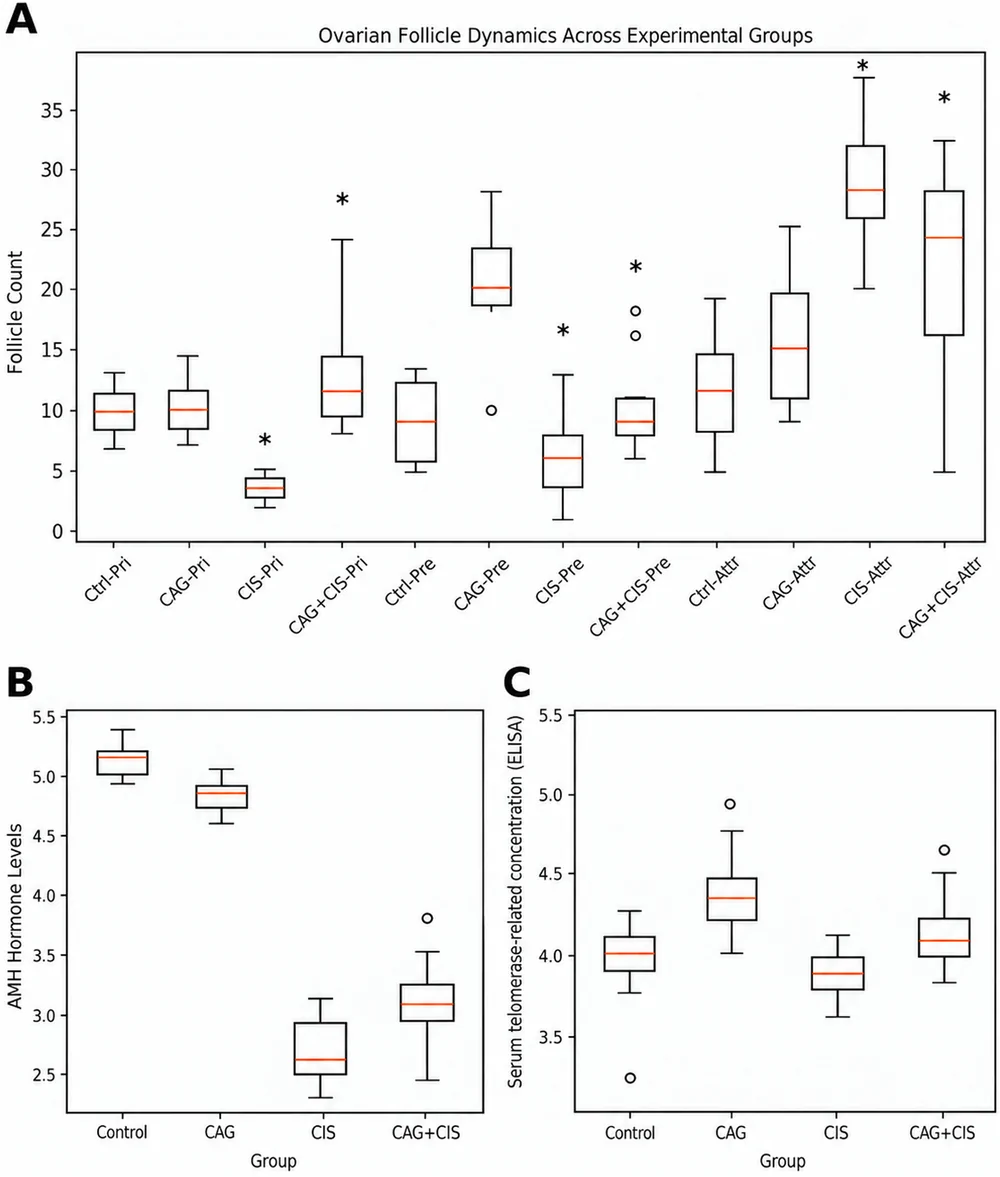
Quantitative assessment of ovarian follicle counts, serum anti‐Müllerian hormone (AMH) levels, and serum telomerase‐related concentrations measured using an ELISA‐based assay among the experimental groups. (A) Numbers of primordial, preantral, and atretic follicles in the control, cycloastragenol (CAG), cisplatin (CIS), and CIS + CAG groups. (B) Serum AMH levels determined by enzyme‐linked immunosorbent assay (ELISA). (C) Serum telomerase‐related concentrations measured using an ELISA‐based assay. Data are presented as individual values with the median and interquartile range. Statistical significance is indicated in the figure. The ELISA‐based serum telomerase‐related measurements should not be interpreted as direct measurements of telomerase activity.

CIS administration induced pronounced ovarian damage, characterized by alterations in the tunica albuginea, degeneration of granulosa cells, and a marked reduction in the primordial follicle pool (Figure 1D). Primordial follicle counts were significantly decreased compared with both the control and CAG groups (Figure 2A, *p* < 0.001 and *p* = 0.015, respectively). Preantral follicles showed extensive oocyte degeneration and increased granulosa cell pyknosis, accompanied by a significant increase in atretic follicles (Figure 2A, *p* < 0.05).

In the CIS + CAG group, degenerative alterations were markedly attenuated. The germinal epithelium and tunica albuginea resembled those of the control group (Figure 1E). Primordial follicle counts were significantly higher than those in the CIS group and were comparable to control values (Figure 2A, *p* < 0.001). Preantral follicle numbers were also significantly increased compared with the CIS group (Figure 2A, *p* < 0.001). Although atretic follicle numbers remained elevated compared with the CAG‐only group (Figure 2A, *p* = 0.031), follicular organization was substantially preserved.

### 3.2 Proliferation Marker Analysis (PCNA Immunohistochemistry)

Strong nuclear PCNA immunoreactivity was observed in oocytes and granulosa cells in the control and CAG groups (Figure 3A–C). PCNA‐positive cell density did not differ significantly between the control and CAG groups (Figure 4D; adjusted *p* = 1.000). CIS treatment markedly reduced the density of PCNA‐positive cells, particularly in preantral follicles, compared with both the control and CAG groups (Figures 3D and 4D; adjusted *p* = 0.008 and *p* = 0.006, respectively).

**Figure 3:**
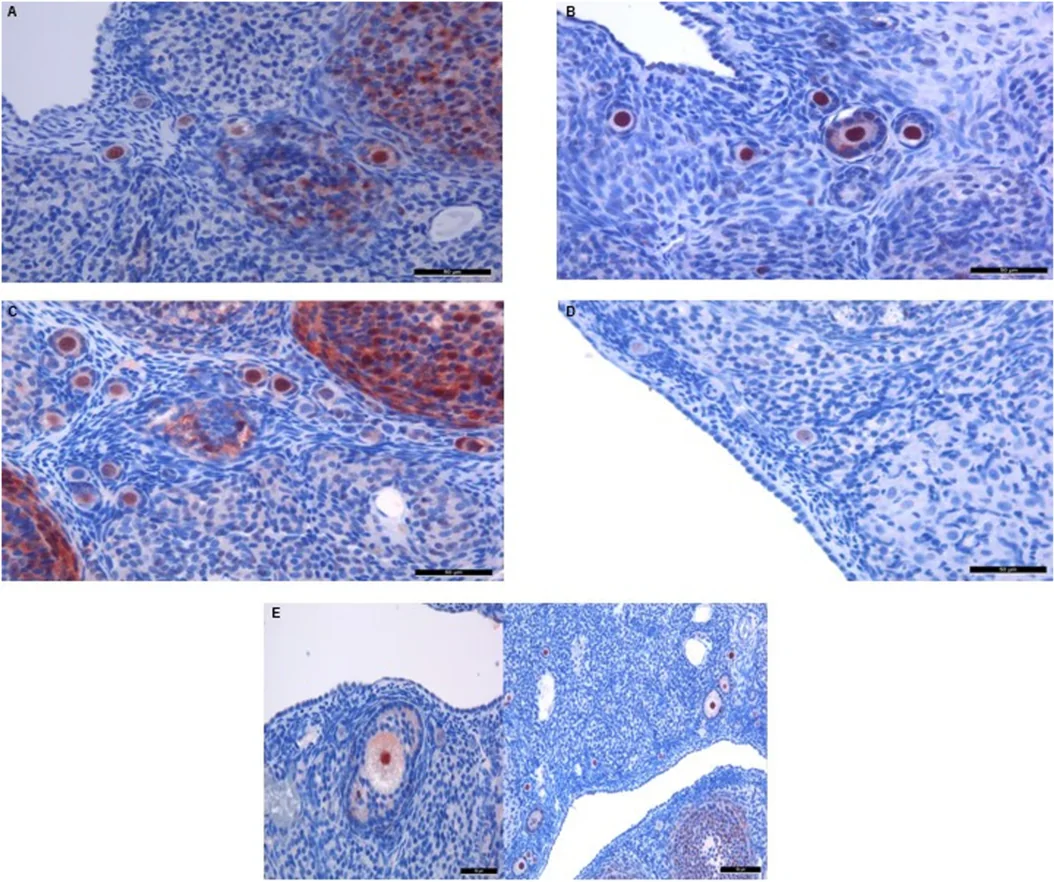
Representative micrographs showing proliferating cell nuclear antigen (PCNA) immunostaining in ovarian sections from the control (vehicle–gavage) group (A), control (vehicle–intraperitoneal DMSO) group (B), cycloastragenol (CAG) group (C), cisplatin (CIS) group (D), and cisplatin + CAG group (E). PCNA‐positive nuclei are visible as brown nuclear staining. Scale bar = 50 μm.

**Figure 4:**
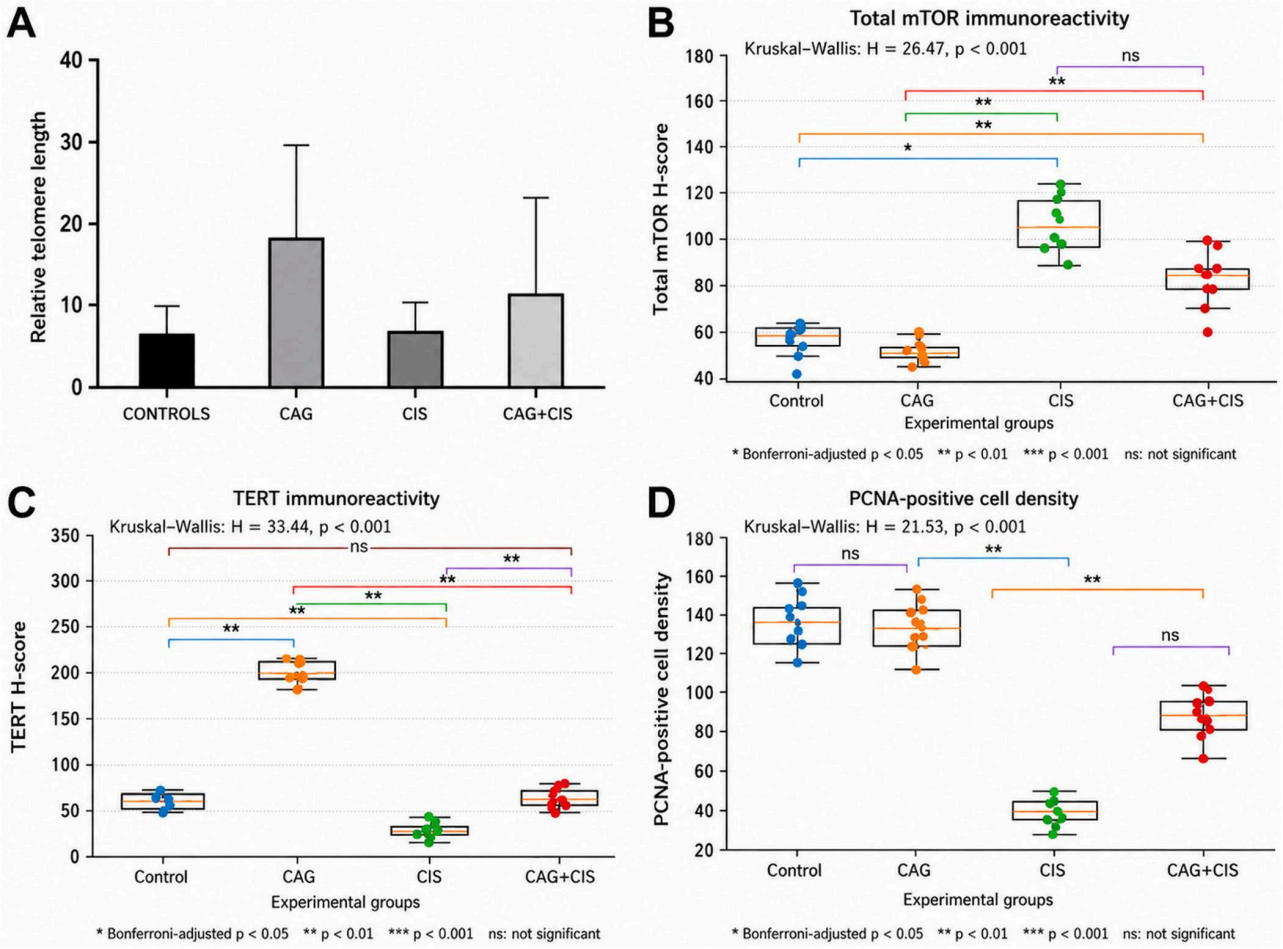
Quantitative assessment of relative telomere length and ovarian immunohistochemical markers among the experimental groups. (A) Relative telomere length measured by qPCR across the experimental groups. (B) Total mammalian target of rapamycin (mTOR) immunoreactivity expressed as *H*‐scores. (C) Telomerase reverse transcriptase (TERT) immunoreactivity expressed as *H*‐scores. (D) Quantification of proliferating cell nuclear antigen (PCNA)‐positive cells. Data are presented as individual values with the median and interquartile range. Statistical significance is indicated in the figure.

In the CIS + CAG group, nuclear PCNA immunoreactivity appeared partially restored in preantral follicles (Figure 3E). However, PCNA‐positive cell density did not differ significantly between the CIS and CIS + CAG groups (Figure 4D; adjusted *p* = 1.000). Some primordial follicles in both CAG‐treated groups remained PCNA‐negative (Figure 3C,E), consistent with maintenance of a non‐proliferative state.

### 3.3 FOXO3a Localization and Expression

Total FOXO3a immunoreactivity was evaluated separately in the nuclear and cytoplasmic compartments (Figures 5 and 6A,B).

Nuclear FOXO3a *H*‐scores differed significantly among the experimental groups (Kruskal–Wallis *H* = 30.20, *p* < 0.001; Figure 6A). Nuclear immunoreactivity was significantly lower in the CIS and CIS + CAG groups than in both the control and CAG groups (Bonferroni‐adjusted *p* = 0.001 for all comparisons). No significant difference was detected between the control and CAG groups (adjusted *p* = 1.000). Although nuclear FOXO3a immunoreactivity was numerically higher in the CIS + CAG group than in the CIS group, this difference did not reach statistical significance (adjusted *p* = 0.410). Consistent with the quantitative findings, prominent nuclear FOXO3a staining was observed in the control and CAG groups, whereas nuclear immunoreactivity was markedly reduced in both CIS‐treated groups (Figure 5A–E).

**Figure 5:**
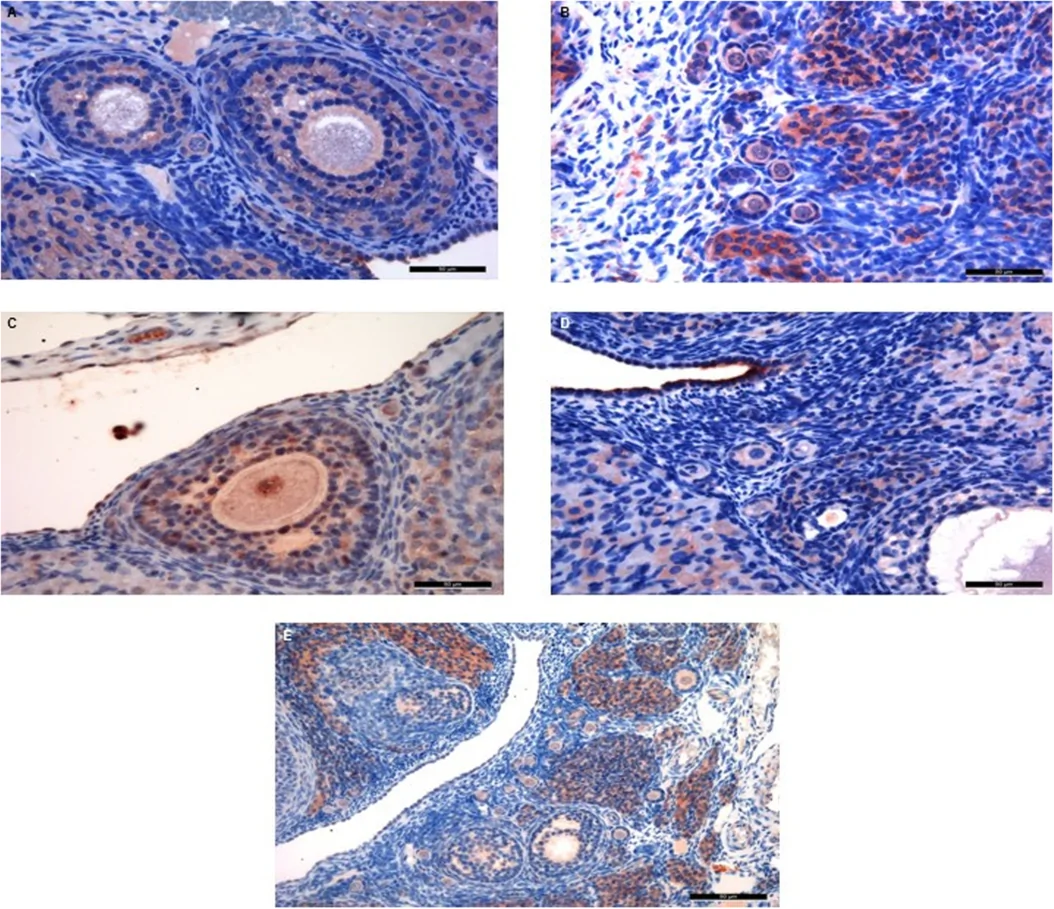
Representative micrographs showing total FOXO3a immunostaining in ovarian sections from the vehicle–gavage control (A), vehicle–intraperitoneal DMSO control (B), cycloastragenol (CAG) (C), cisplatin (CIS) (D), and CIS + CAG (E) groups. Prominent nuclear and cytoplasmic FOXO3a immunoreactivity was observed in oocytes and granulosa cells in the control and CAG groups. Cisplatin exposure was associated with a marked reduction in both nuclear and cytoplasmic FOXO3a staining. In the CIS + CAG group, cytoplasmic immunoreactivity appeared partially restored compared with the CIS group, whereas nuclear staining remained reduced. Scale bar = 50 μm.

Cytoplasmic FOXO3a *H*‐scores also differed significantly among the groups (Kruskal–Wallis *H* = 33.92, *p* < 0.001; Figure 6B). Cytoplasmic immunoreactivity was significantly lower in the CIS group than in the control and CAG groups (adjusted *p* = 0.001 for both comparisons). CAG co‐treatment significantly increased cytoplasmic FOXO3a immunoreactivity compared with CIS alone (adjusted *p* = 0.003). However, cytoplasmic *H*‐scores in the CIS + CAG group remained significantly lower than those in the control and CAG groups (adjusted *p* = 0.001 for both comparisons). No significant difference was observed between the control and CAG groups (adjusted *p* = 0.113). Morphologically, cytoplasmic FOXO3a staining was markedly reduced following CIS exposure and appeared partially restored in the CIS + CAG group (Figure 5D,E).

**Figure 6:**
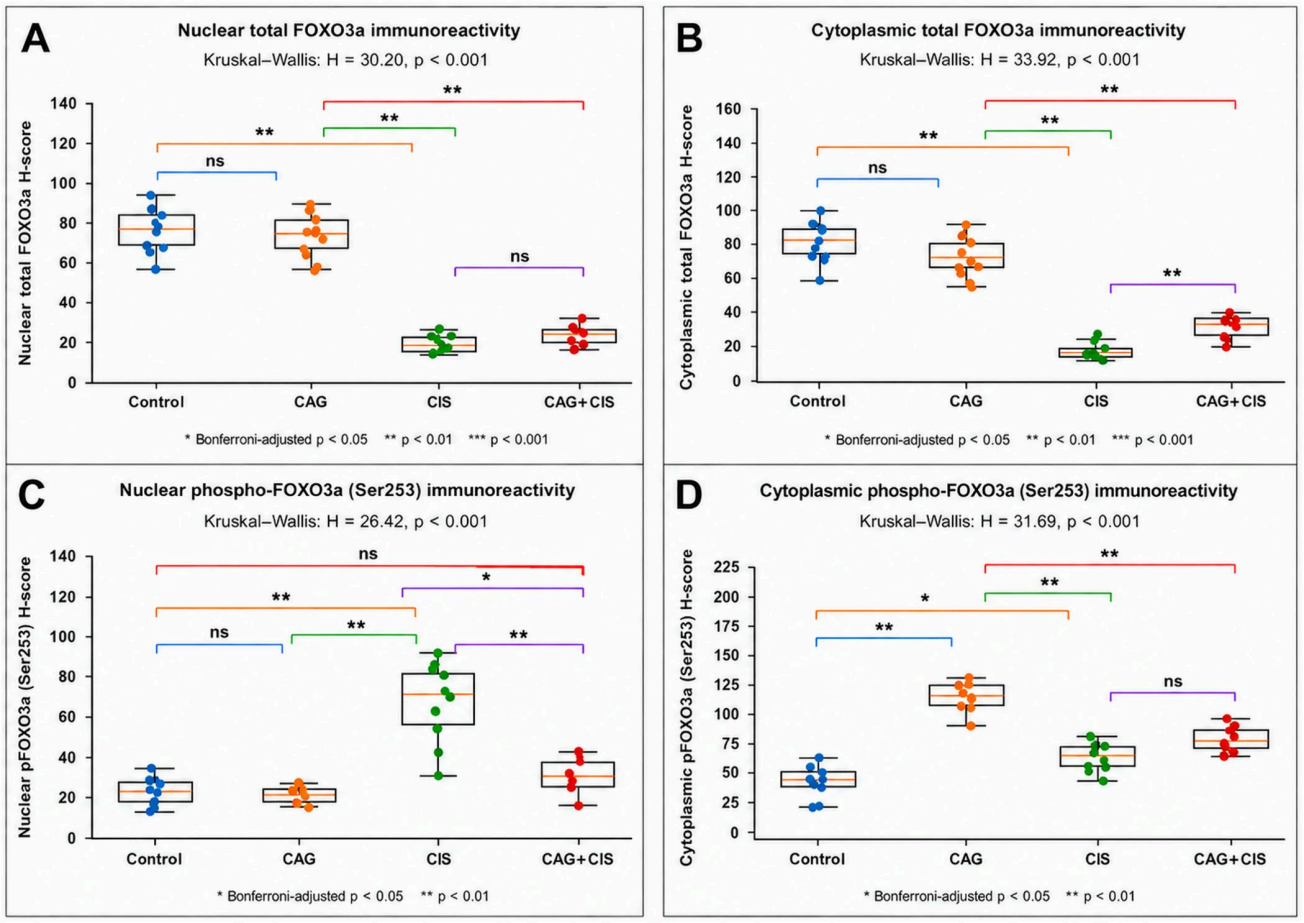
Quantitative assessment of total FOXO3a and phospho‐FOXO3a (Ser253) immunoreactivity in the nuclear and cytoplasmic compartments of ovarian tissue. (A) Nuclear total FOXO3a *H*‐scores. (B) Cytoplasmic total FOXO3a *H*‐scores. (C) Nuclear phospho‐FOXO3a (Ser253) *H*‐scores. (D) Cytoplasmic phospho‐FOXO3a (Ser253) *H*‐scores. Data are presented as individual values with the median and interquartile range. Statistical significance is indicated in the figure.

Overall, CAG co‐treatment was associated with a significant partial restoration of cytoplasmic total FOXO3a immunoreactivity, whereas the numerical increase in nuclear FOXO3a immunoreactivity did not reach statistical significance.

### 3.4 Phospho‐FOXO3a (Ser253) Expression and Subcellular Localization

Phospho‐FOXO3a (Ser253) immunoreactivity was evaluated separately in the nuclear and cytoplasmic compartments. *H*‐scores differed significantly among the experimental groups for both nuclear staining (Kruskal–Wallis *H* = 26.42, *p* < 0.001; Figure 6C) and cytoplasmic staining (Kruskal–Wallis *H* = 31.69, *p* < 0.001; Figure 6D).

Nuclear pFOXO3a immunoreactivity was most prominent in the CIS group, whereas relatively low nuclear staining was observed in the control and CAG groups (Figure 7A–D). Nuclear staining appeared reduced in the CIS + CAG group compared with the CIS group (Figure 7D,E). Quantitative analysis showed that nuclear pFOXO3a *H*‐scores were significantly higher in the CIS group than in the control, CAG, and CIS + CAG groups (Bonferroni‐adjusted *p* = 0.002, *p* = 0.001, and *p* = 0.006, respectively; Figure 6C). Nuclear immunoreactivity was also significantly higher in the CIS + CAG group than in the CAG group (adjusted *p* = 0.011). No significant difference was detected between the CIS + CAG and control groups (adjusted *p* = 0.122) or between the control and CAG groups (adjusted *p* = 1.000).

**Figure 7:**
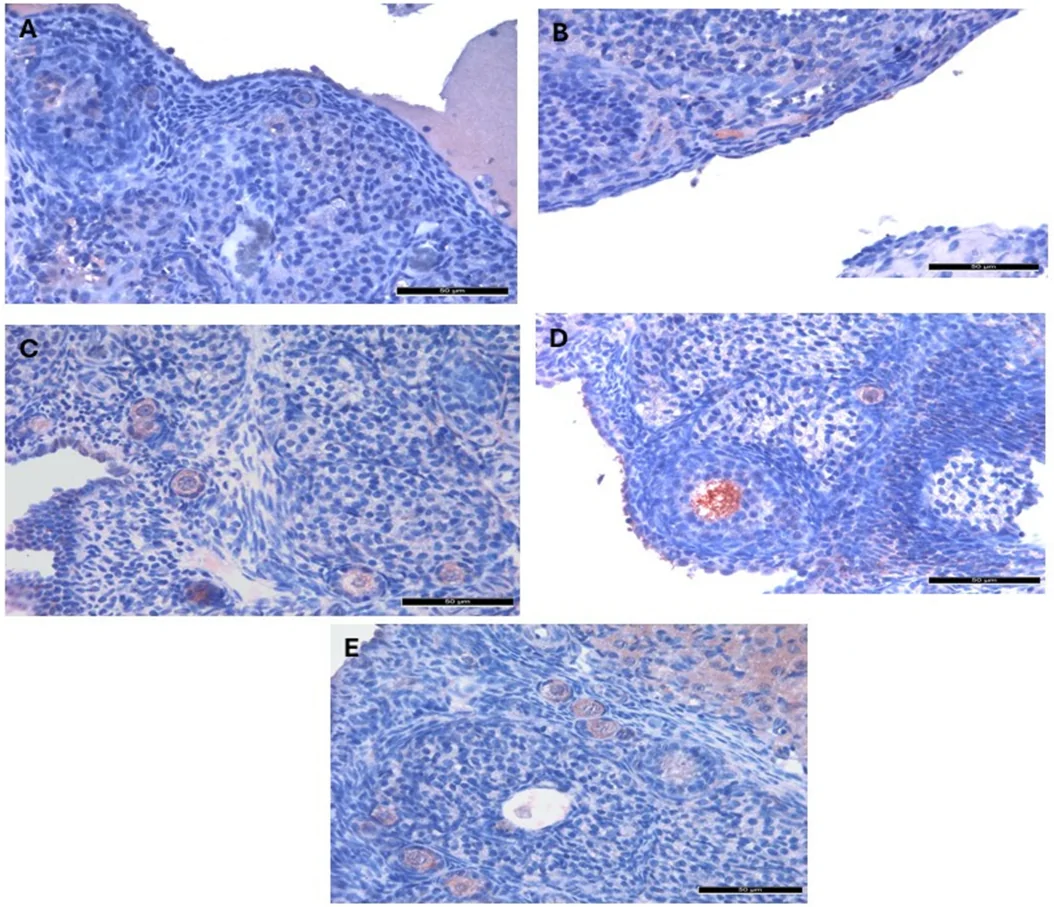
Representative micrographs showing phospho‐FOXO3a (Ser253) immunostaining in ovarian sections from the vehicle–gavage control (A), vehicle–intraperitoneal DMSO control (B), cycloastragenol (CAG) (C), cisplatin (CIS) (D), and CIS + CAG (E) groups. Cytoplasmic pFOXO3a immunoreactivity was relatively low in the control groups and markedly increased in the CAG group. Cisplatin exposure was associated with prominent nuclear pFOXO3a staining, whereas nuclear immunoreactivity was visibly reduced in the CIS + CAG group compared with the CIS group. Cytoplasmic staining remained evident in both cisplatin‐treated groups. Scale bar = 50 μm.

Cytoplasmic pFOXO3a immunoreactivity was relatively low in the control groups and markedly increased in the CAG group (Figure 7A–C). Cytoplasmic *H*‐scores were significantly higher in the CAG group than in the control, CIS, and CIS + CAG groups (adjusted *p* = 0.001 for all comparisons; Figure 6D). Cytoplasmic immunoreactivity was also significantly higher in the CIS and CIS + CAG groups than in the control group (adjusted *p* = 0.043 and *p* = 0.001, respectively). No significant difference was detected between the CIS and CIS + CAG groups (adjusted *p* = 0.321; Figure 6D).

Overall, CIS exposure was associated with increased nuclear pFOXO3a immunoreactivity. CAG co‐treatment significantly reduced nuclear pFOXO3a staining compared with CIS alone but did not significantly alter cytoplasmic pFOXO3a immunoreactivity. CAG administration alone was associated with a marked increase in cytoplasmic pFOXO3a staining.

### 3.5 mTOR Expression and Subcellular Localization

Total mTOR immunoreactivity differed significantly among the experimental groups (Kruskal–Wallis *H* = 26.47, *p* < 0.001; Figure 4B). Total mTOR *H*‐scores were significantly higher in the CIS group than in the control and CAG groups (Bonferroni‐adjusted *p* = 0.012 and *p* = 0.007, respectively). The CIS + CAG group also exhibited significantly higher total mTOR immunoreactivity than the control and CAG groups (adjusted *p* = 0.003 and *p* = 0.001, respectively). No significant difference was detected between the control and CAG groups (adjusted *p* = 0.315). Although the total mTOR *H*‐score was lower in the CIS + CAG group than in the CIS group, this difference did not reach statistical significance after Bonferroni correction (adjusted *p* = 0.060; Figure 4B).

Morphologically, punctate nuclear mTOR immunoreactivity was visible in the control groups and appeared more prominent in the CAG group (Figure 8A–C). CIS exposure was associated with increased cytoplasmic mTOR staining and an apparent reduction in the punctate nuclear pattern (Figure 8D). In the CIS + CAG group, punctate nuclear mTOR‐positive structures were again visible (Figure 8E). Because this nuclear staining pattern was not quantified as an independent localization endpoint, these findings should be interpreted solely as qualitative morphological observations.

**Figure 8:**
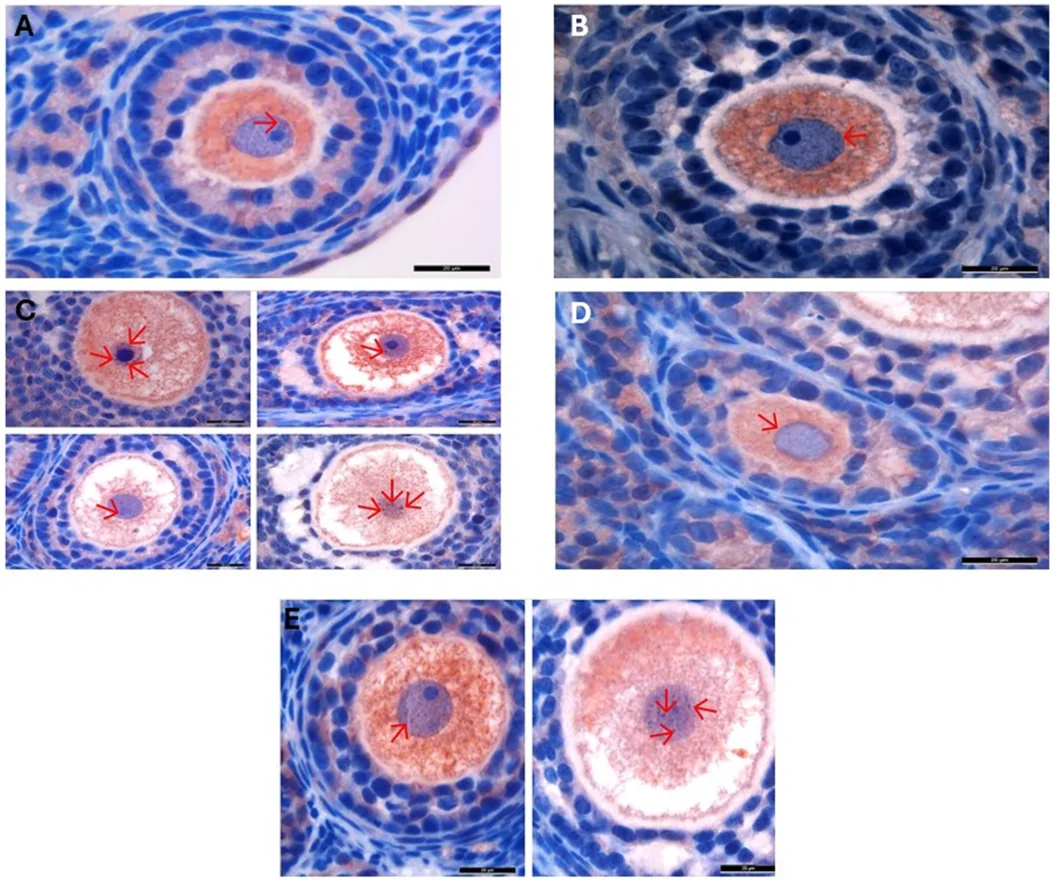
Representative micrographs showing total mTOR immunostaining in ovarian sections from the vehicle–gavage control (A), vehicle–intraperitoneal DMSO control (B), cycloastragenol (CAG) (C), cisplatin (CIS) (D), and CIS + CAG (E) groups. Punctate nuclear mTOR immunoreactivity was visible in the control groups and was particularly prominent in the CAG group. The punctate nuclear staining pattern represents a qualitative morphological observation and was not quantified as an independent localization endpoint. Because only total mTOR immunoreactivity was evaluated, these observations should not be interpreted as direct evidence of mTOR pathway activation. Scale bar = 20 μm.

Because total rather than phosphorylated mTOR was evaluated, these findings reflect changes in mTOR immunoreactivity and subcellular distribution and should not be interpreted as direct evidence of mTOR pathway activation.

### 3.6 Apoptotic Activity (Caspase‐3 Immunohistochemistry)

Caspase‐3 immunoreactivity differed significantly among the experimental groups (Kruskal–Wallis *H* = 31.39, *p* < 0.001; Figure 10D). Caspase‐3 *H*‐scores were significantly higher in the CIS group than in the control and CAG groups (Bonferroni‐adjusted *p* = 0.001 for both comparisons). The CIS + CAG group also exhibited significantly higher caspase‐3 immunoreactivity than the control and CAG groups (adjusted *p* = 0.001 for both comparisons). No significant difference was detected between the control and CAG groups (adjusted *p* = 1.000). Although the caspase‐3 *H*‐score was numerically lower in the CIS + CAG group than in the CIS group, this difference did not reach statistical significance after Bonferroni correction (adjusted *p* = 0.054; Figure 10D).

Morphologically, weak cytoplasmic caspase‐3 immunoreactivity was observed in the control and CAG groups (Figure 9A–C). CIS exposure was associated with markedly increased cytoplasmic staining, predominantly in oocytes and granulosa cells (Figure 9D). In the CIS + CAG group, caspase‐3 immunoreactivity appeared lower than in the CIS group but remained more prominent than in the control and CAG groups (Figure 9E).

**Figure 9:**
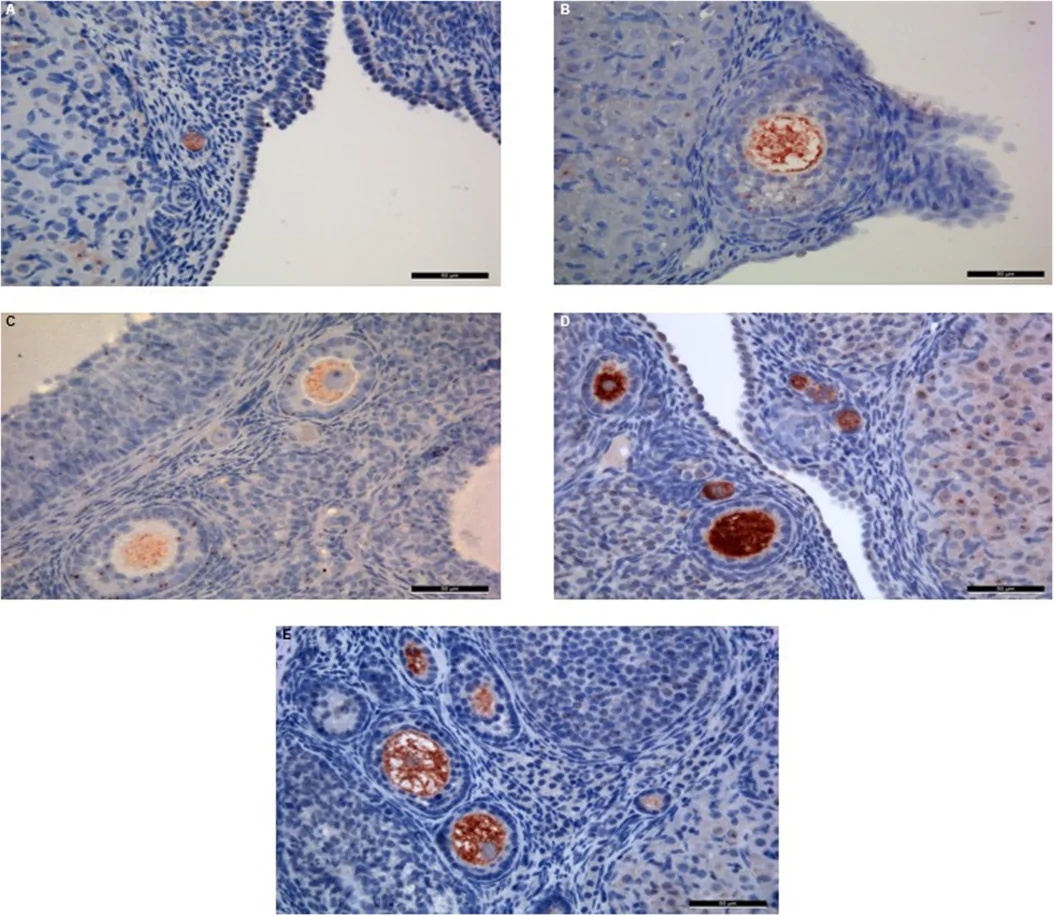
Representative micrographs showing caspase‐3 immunostaining in ovarian sections from the vehicle–gavage control (A), vehicle–intraperitoneal DMSO control (B), cycloastragenol (CAG) (C), cisplatin (CIS) (D), and CIS + CAG (E) groups. Weak cytoplasmic caspase‐3 immunoreactivity was observed in the control and CAG groups. Cisplatin exposure was associated with markedly increased cytoplasmic staining, predominantly in oocytes and granulosa cells. In the CIS + CAG group, caspase‐3 immunoreactivity appeared lower than in the CIS group but remained more prominent than in the control and CAG groups. Sections were counterstained with Harris hematoxylin. Scale bar = 50 μm.

Overall, CAG co‐treatment was associated with a numerical reduction in CIS‐induced caspase‐3 immunoreactivity. However, because this difference was not statistically significant, the finding should be interpreted as a decreasing trend rather than evidence of a significant reduction in caspase‐3‐mediated apoptotic activity.

### 3.7 BAX and BCL‐2 Immunoreactivity

BAX and BCL‐2 immunoreactivity showed opposing patterns among the experimental groups, indicating a CIS‐associated shift in the ovarian apoptotic balance.

BAX *H*‐scores differed significantly among the groups (Kruskal–Wallis *H* = 35.30, *p* < 0.001; Figure 10A). The CIS group exhibited significantly higher BAX immunoreactivity than the control, CAG, and CIS + CAG groups (Bonferroni‐adjusted *p* = 0.001 for all comparisons). Although CAG co‐treatment significantly reduced BAX immunoreactivity compared with CIS alone, the CIS + CAG group remained significantly higher than the control and CAG groups (adjusted *p* = 0.001 for both comparisons). The CAG group also showed lower BAX immunoreactivity than the control group (adjusted *p* = 0.016). Morphologically, weak BAX staining was observed in the control and CAG groups (Figure 11A–C), whereas intense immunoreactivity was evident following CIS exposure (Figure 11D). In the CIS + CAG group, BAX staining was visibly reduced compared with the CIS group but remained more prominent than in the control and CAG groups (Figure 11E).

**Figure 10:**
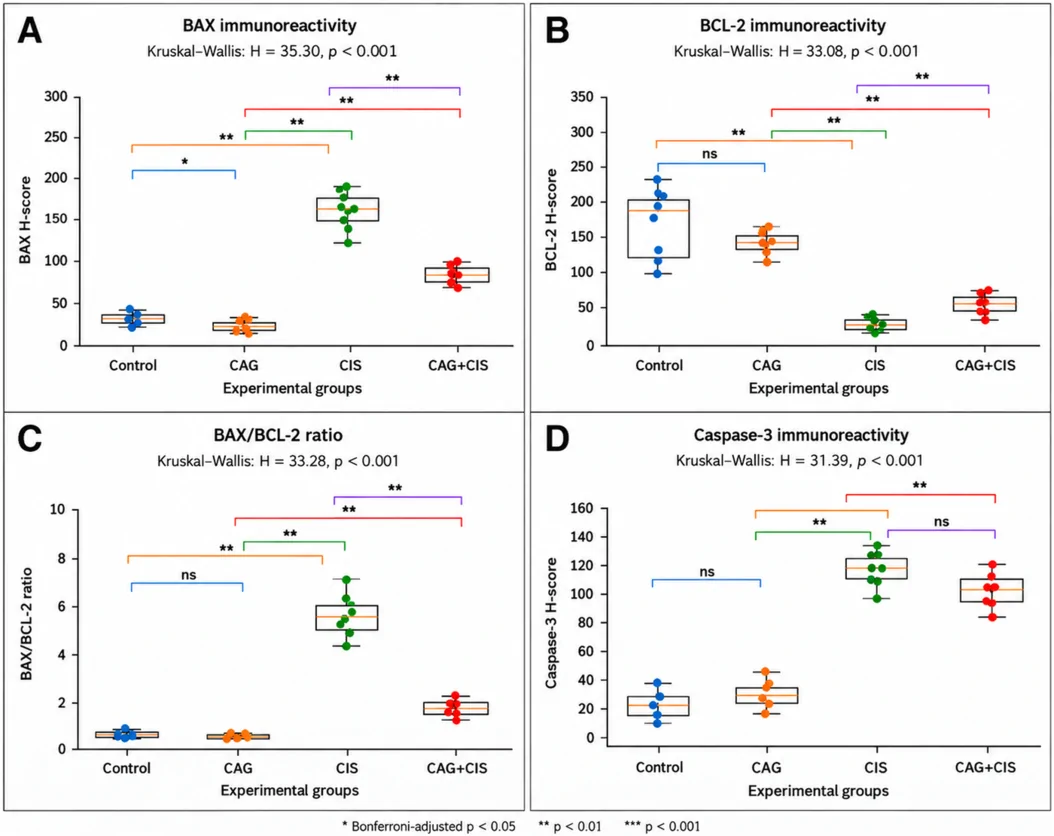
Quantitative assessment of apoptosis‐related immunohistochemical markers in ovarian tissue across the experimental groups. (A) BAX immunoreactivity expressed as *H*‐scores. (B) BCL‐2 immunoreactivity expressed as *H*‐scores. (C) BAX/BCL‐2 *H*‐score ratio. (D) Caspase‐3 immunoreactivity expressed as *H*‐scores. Data are presented as individual values with the median and interquartile range. Statistical significance is indicated in the figure.

**Figure 11:**
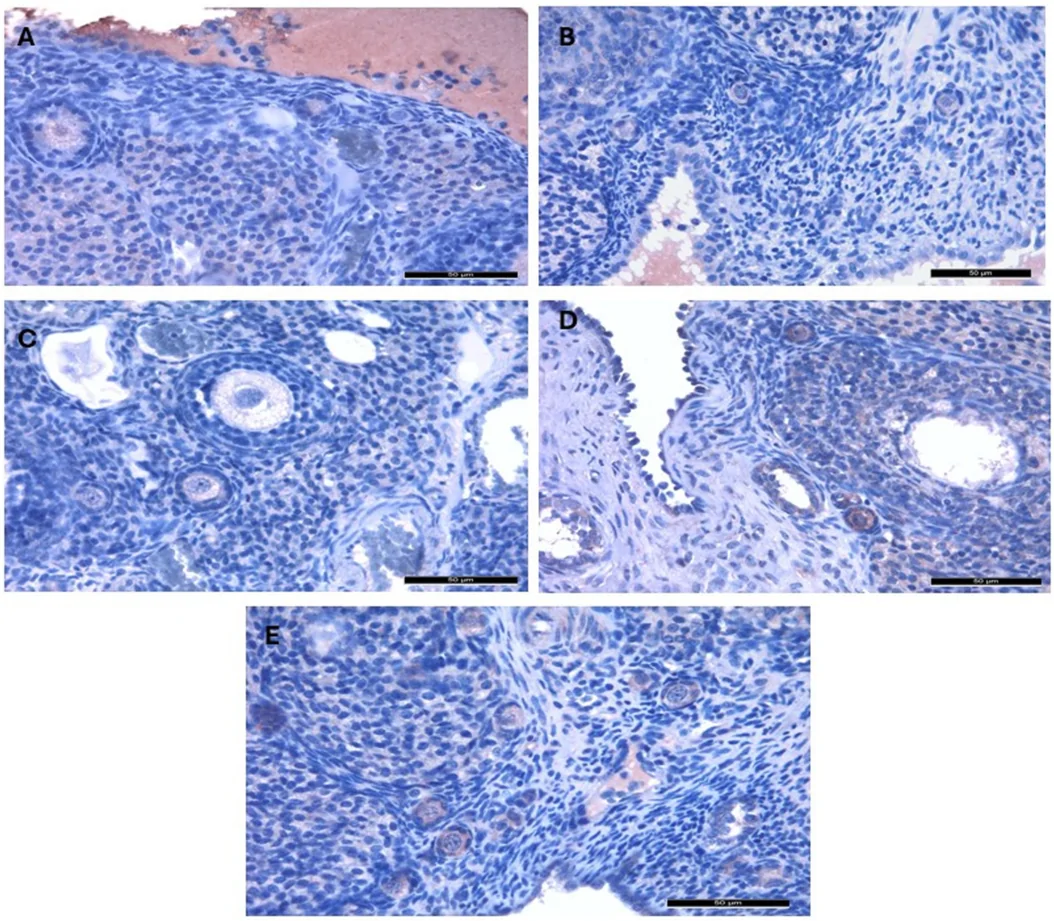
Representative micrographs showing BAX immunostaining in ovarian sections from the vehicle–gavage control (A), vehicle–intraperitoneal DMSO control (B), cycloastragenol (CAG) (C), cisplatin (CIS) (D), and CIS + CAG (E) groups. Weak BAX immunoreactivity was observed in the control groups and was further reduced in the CAG group. Cisplatin exposure was associated with markedly increased BAX staining, predominantly in oocytes and granulosa cells. In the CIS + CAG group, BAX immunoreactivity was visibly reduced compared with the CIS group but remained more prominent than in the control and CAG groups. Sections were counterstained with Harris hematoxylin. Scale bar = 50 μm.

BCL‐2 *H*‐scores also differed significantly among the groups (Kruskal–Wallis *H* = 33.08, *p* < 0.001; Figure 10B). The CIS group exhibited significantly lower BCL‐2 immunoreactivity than the control, CAG, and CIS + CAG groups (Bonferroni‐adjusted *p* = 0.001 for all comparisons). CAG co‐treatment significantly increased BCL‐2 immunoreactivity compared with CIS alone; however, the CIS + CAG group remained significantly lower than the control and CAG groups (adjusted *p* = 0.001 for both comparisons). No significant difference was detected between the control and CAG groups (adjusted *p* = 1.000). Prominent BCL‐2 staining was observed in the control and CAG groups (Figure 12A–C), whereas CIS markedly reduced immunoreactivity (Figure 12D). Partial restoration of BCL‐2 staining was evident in the CIS + CAG group (Figure 12E).

**Figure 12:**
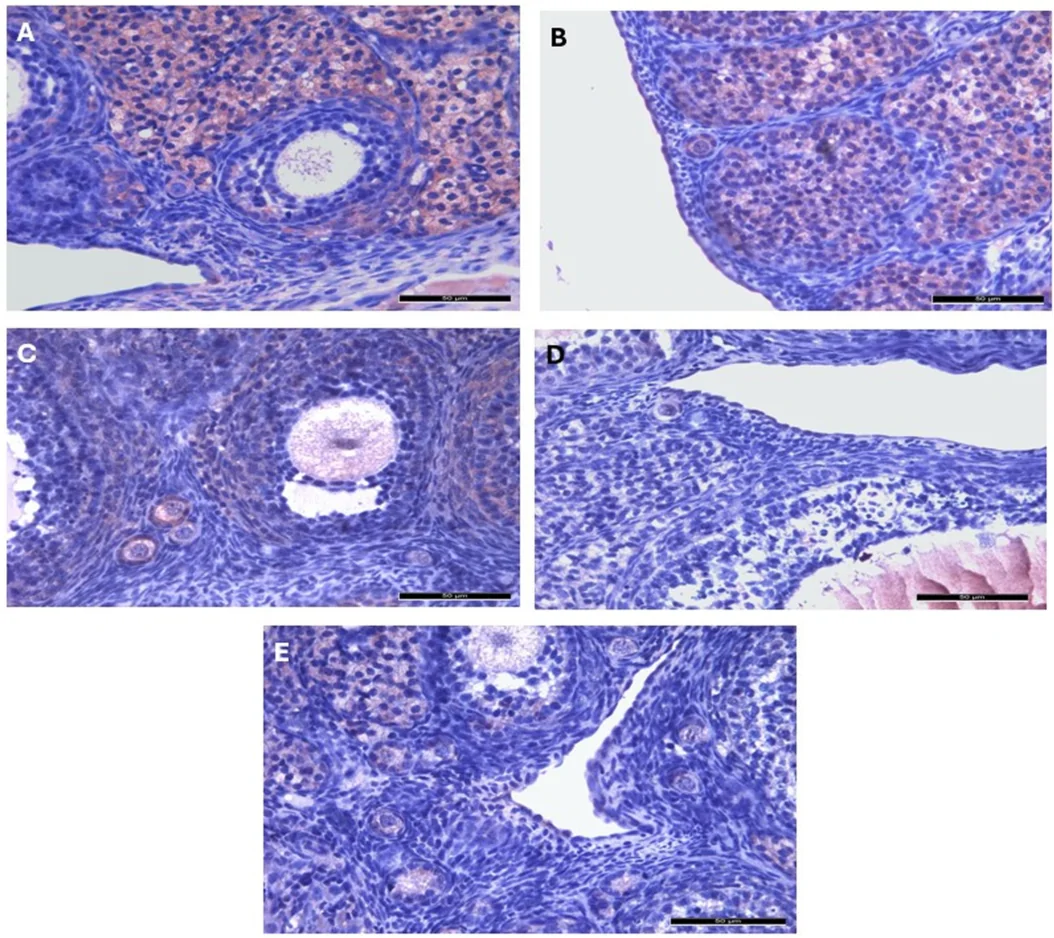
Representative micrographs showing BCL‐2 immunostaining in ovarian sections from the vehicle–gavage control (A), vehicle–intraperitoneal DMSO control (B), cycloastragenol (CAG) (C), cisplatin (CIS) (D), and CIS + CAG (E) groups. Prominent BCL‐2 immunoreactivity was observed in the control and CAG groups, whereas cisplatin exposure markedly reduced staining, particularly in oocytes and granulosa cells. In the CIS + CAG group, BCL‐2 immunoreactivity was visibly increased compared with the CIS group but remained lower than in the control and CAG groups. Sections were counterstained with Harris hematoxylin. Scale bar = 50 μm.

Consistent with these opposing changes, the BAX/BCL‐2 ratio differed significantly among the groups (Kruskal–Wallis *H* = 33.28, *p* < 0.001; Figure 10C). The ratio was significantly higher in the CIS group than in all other groups (Bonferroni‐adjusted *p* = 0.001 for all comparisons). CAG co‐treatment significantly reduced the ratio compared with CIS alone, although it remained significantly higher than in the control and CAG groups (adjusted *p* = 0.001 for both comparisons). No significant difference was observed between the control and CAG groups (adjusted *p* = 1.000).

Overall, CIS shifted the ovarian apoptotic balance toward a pro‐apoptotic profile, whereas CAG co‐treatment significantly attenuated, but did not completely reverse, these alterations.

### 3.8 TERT Immunoreactivity

Ovarian TERT immunoreactivity differed significantly among the experimental groups (Kruskal–Wallis *H* = 33.44, *p* < 0.001; Figure 4C). TERT *H*‐scores were significantly higher in the CAG group than in the control, CIS, and CIS + CAG groups (Bonferroni‐adjusted *p* = 0.001 for all comparisons). CIS treatment significantly reduced TERT immunoreactivity compared with the control group (adjusted *p* = 0.001), whereas CAG co‐treatment significantly increased TERT immunoreactivity compared with CIS alone (adjusted *p* = 0.001). No significant difference was detected between the control and CIS + CAG groups (adjusted *p* = 1.000), indicating restoration of TERT immunoreactivity to a level comparable with that of the control group.

Morphologically, moderate TERT immunoreactivity was observed in the control groups, whereas markedly stronger staining was evident in the CAG group (Figure 13A–C). CIS exposure reduced TERT staining (Figure 13D), while the CIS + CAG group showed increased immunoreactivity compared with CIS alone and a staining pattern comparable with that of the control groups (Figure 13E).

**Figure 13:**
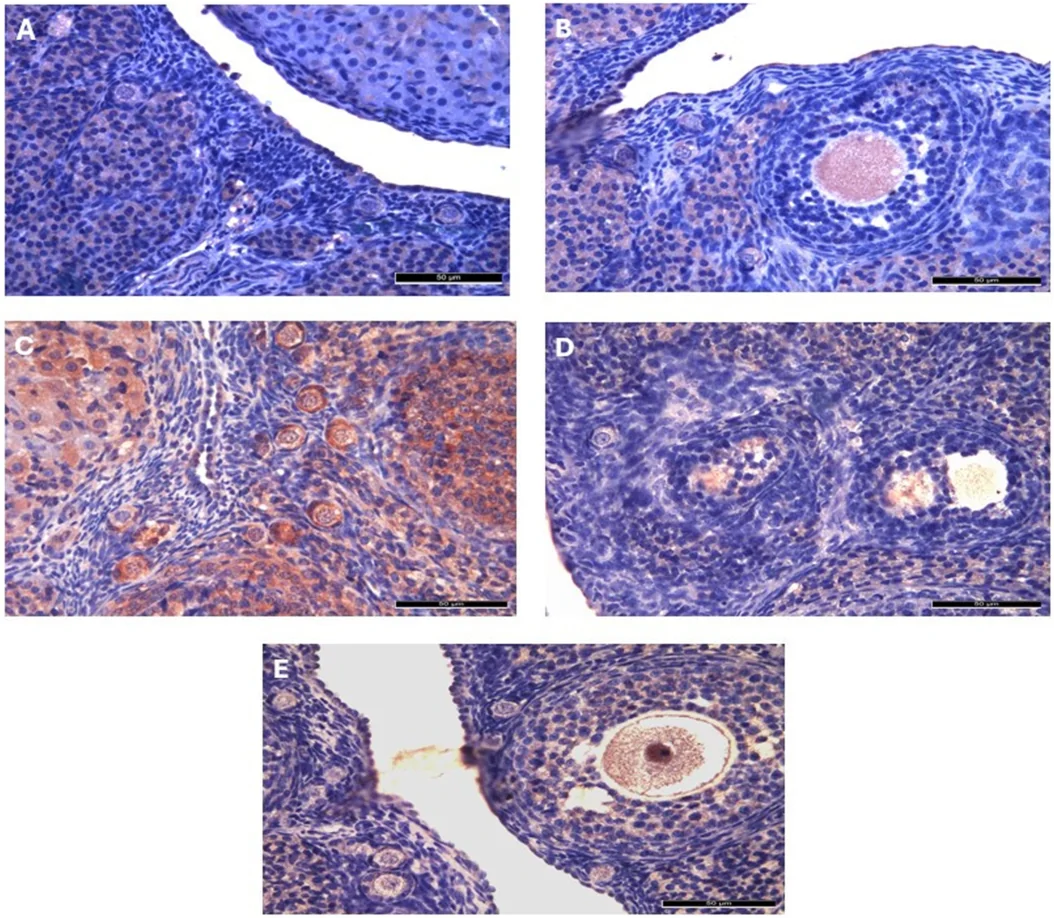
Representative micrographs showing telomerase reverse transcriptase (TERT) immunostaining in ovarian sections from the vehicle–gavage control (A), vehicle–intraperitoneal DMSO control (B), cycloastragenol (CAG) (C), cisplatin (CIS) (D), and CIS + CAG (E) groups. Moderate TERT immunoreactivity was observed in the control groups, whereas the CAG group exhibited markedly enhanced staining. Cisplatin exposure was associated with reduced TERT immunoreactivity. In the CIS + CAG group, TERT staining was increased compared with the CIS group and appeared comparable to that observed in the control groups. Sections were counterstained with Harris hematoxylin. Scale bar = 50 μm.

Overall, CAG administration was associated with increased ovarian TERT immunoreactivity and reversal of the CIS‐associated reduction. However, TERT immunohistochemistry reflects protein immunoreactivity and should not be interpreted as a direct measurement of telomerase activity.

### 3.9 Serum AMH Levels

Serum AMH levels differed among the experimental groups. AMH was significantly lower in the CIS group than in the control and CAG groups (Figure 2B; adjusted *p* = 0.030 and *p* < 0.001, respectively). Although serum AMH levels were numerically higher in the CIS + CAG group than in the CIS group, this difference did not reach statistical significance (adjusted *p* = 0.070).

These findings indicate that CIS was associated with reduced serum AMH, whereas CAG co‐treatment produced a numerical, but not statistically significant, increase.

### 3.10 Serum Telomerase‐Related ELISA Measurements

Serum telomerase‐related concentrations differed among the experimental groups. Concentrations were significantly higher in the CAG group than in the control group (Figure 2C; adjusted *p* = 0.012). CIS treatment was associated with a numerical reduction compared with the control group, although this difference was not statistically significant (adjusted *p* = 0.100). Serum telomerase‐related concentrations were significantly higher in the CIS + CAG group than in the CIS group (adjusted *p* = 0.017), approaching those observed in the control group.

These findings indicate that CAG administration was associated with increased serum telomerase‐related concentrations. However, because the measurement was ELISA‐based, it should not be interpreted as a direct measurement of telomerase activity.

### 3.11 Telomere Length Analysis

Relative telomere length was evaluated in ovarian tissue to determine whether CIS exposure or CAG treatment was associated with measurable changes in telomere status during the experimental period. Although minor numerical differences were observed among the groups, the overall group comparison did not reach statistical significance (Figure 4A; Kruskal–Wallis *p* > 0.05). Therefore, neither CIS treatment nor CAG administration produced a statistically demonstrable change in relative ovarian telomere length under the conditions and duration of the present study.

These findings indicate that the observed histological, apoptotic, and immunohistochemical alterations were not accompanied by detectable changes in relative telomere length. Accordingly, the telomere‐length results should be interpreted as unchanged across the experimental groups rather than as evidence of a treatment‐related increasing or decreasing trend.

## 4 Discussion

The present study demonstrated that CAG partially attenuated CIS‐induced ovarian injury in rats. CIS exposure was associated with depletion of the primordial follicle pool, increased follicular atresia, reduced proliferative activity, disruption of the ovarian apoptotic balance, decreased TERT immunoreactivity, and alterations in the expression and subcellular distribution of FOXO3a and mTOR. CAG co‐treatment significantly improved BAX and BCL‐2 immunoreactivity, the BAX/BCL‐2 ratio, cytoplasmic total FOXO3a, nuclear phospho‐FOXO3a, and ovarian TERT immunoreactivity. However, PCNA‐positive cell density, nuclear total FOXO3a, caspase‐3, total mTOR, and serum AMH did not show statistically significant recovery compared with CIS alone, indicating partial rather than complete protection.

The histological findings were consistent with the established gonadotoxic effects of CIS, including granulosa‐cell degeneration, follicular atresia, and depletion of the primordial follicle pool [1, 2]. CIS may also promote abnormal recruitment of dormant primordial follicles through dysregulation of PTEN/AKT/FOXO3a signaling [3, 4]. In the present study, primordial follicle counts were higher and degenerative alterations were less pronounced in the CIS + CAG group than in the CIS group, suggesting attenuation of structural ovarian injury. Nevertheless, preservation of follicular morphology and follicle numbers cannot be considered direct evidence of preserved reproductive function because estrous cyclicity, ovulation, pregnancy rates, and offspring outcomes were not evaluated.

The apoptosis‐related findings provided the most consistent evidence of CAG‐associated protection. CIS markedly increased BAX immunoreactivity, reduced BCL‐2 immunoreactivity, and elevated the BAX/BCL‐2 ratio, indicating a shift toward mitochondrial pro‐apoptotic signaling [14, 15, 16]. CAG co‐treatment significantly attenuated each of these alterations, although the values did not completely return to control levels. Caspase‐3 immunoreactivity was numerically lower in the CIS + CAG group than in the CIS group, but the difference did not reach statistical significance after Bonferroni correction. Collectively, these findings indicate improvement of the ovarian apoptotic balance rather than complete inhibition of CIS‐induced apoptotic activity.

CIS exposure also significantly reduced PCNA‐positive cell density, particularly in preantral follicles. Although PCNA‐positive cell density was numerically higher following CAG co‐treatment, the difference between the CIS and CIS + CAG groups was not statistically significant. Therefore, the PCNA findings provide only limited evidence of preservation of proliferative activity and should not be interpreted as restoration of follicular cell proliferation.

FOXO3a plays an essential role in maintaining primordial follicle dormancy [6]. In the present study, CIS markedly reduced both nuclear and cytoplasmic total FOXO3a immunoreactivity. CAG co‐treatment significantly increased cytoplasmic total FOXO3a, whereas the numerical increase in nuclear total FOXO3a did not reach statistical significance. Phospho‐FOXO3a (Ser253) exhibited a distinct compartment‐specific pattern. Nuclear pFOXO3a immunoreactivity was highest in the CIS group and was significantly reduced following CAG co‐treatment. By contrast, cytoplasmic pFOXO3a immunoreactivity did not differ significantly between the CIS and CIS + CAG groups, while CAG administration alone was associated with a marked increase in cytoplasmic pFOXO3a staining. Because FOXO3a function depends on its phosphorylation status and subcellular localization, these findings suggest treatment‐associated modulation of CIS‐induced FOXO3a dysregulation [3, 4, 6, 7, 8]. However, immunohistochemical localization alone cannot demonstrate restoration of FOXO3a transcriptional activity or maintenance of primordial follicle quiescence.

Total mTOR immunoreactivity was significantly increased following CIS exposure. Morphologically, punctate nuclear mTOR immunoreactivity was visible in the control groups and was particularly prominent in the CAG group. CIS exposure was associated with increased cytoplasmic staining and disruption of this punctate nuclear pattern. In the CIS + CAG group, cytoplasmic staining appeared reduced, and punctate nuclear mTOR‐positive structures were again visible. However, because the punctate nuclear pattern was not quantified independently, this finding should be interpreted as a qualitative morphological observation rather than quantitative evidence of altered nuclear mTOR localization. Nevertheless, the reduction in total mTOR *H*‐scores between the CIS and CIS + CAG groups did not reach statistical significance after Bonferroni correction. Because only total mTOR was evaluated, these observations reflect treatment‐associated alterations in mTOR immunoreactivity and subcellular distribution rather than direct evidence of pathway activation. Furthermore, the present findings cannot distinguish between mTORC1‐ and mTORC2‐related signaling [9, 10, 11, 12, 13].

Ovarian TERT immunoreactivity was highest in the CAG group and was significantly reduced by CIS. CAG co‐treatment significantly increased TERT immunoreactivity compared with CIS alone and restored the *H*‐score to a level comparable with that of the control group. These findings are consistent with previous studies associating CAG with enhanced telomerase‐related activity and TERT‐associated responses [20, 21, 22]. In addition, experimental studies have linked constitutive TERT expression with increased β‐Klotho expression and attenuation of age‐related deterioration in early bovine embryos [27]. However, immunohistochemical TERT staining does not directly measure enzymatic telomerase activity. Similarly, serum telomerase measurements obtained using an ELISA‐based assay may not accurately reflect local ovarian telomerase activity. Therefore, the present findings support an association between CAG treatment and ovarian TERT immunoreactivity but do not establish that the observed protection was telomerase dependent.

Relative ovarian telomere length did not differ significantly among the experimental groups. The 15‐day experimental period may have been insufficient to produce measurable telomere‐length alterations. In addition, tissue heterogeneity and the analytical limitations of relative telomere qPCR may reduce sensitivity for detecting subtle treatment‐associated differences [28]. The absence of a measurable change in telomere length does not exclude possible telomere‐independent functions of TERT. TERT has been implicated in mitochondrial homeostasis, oxidative‐stress responses, DNA‐damage regulation, and apoptosis, although these mechanisms were not directly examined in the present study [19, 20].

Serum AMH was significantly reduced following CIS exposure. Although AMH levels were numerically higher in the CIS + CAG group than in the CIS group, this difference was not statistically significant. Accordingly, the AMH result should be interpreted as a non‐significant recovery trend rather than evidence that CAG preserved ovarian reserve [29, 30]. This distinction is important because serum AMH is an indirect marker of the follicular pool and cannot independently demonstrate preservation of fertility or long‐term ovarian function.

In addition to its telomerase‐associated actions, CAG has been linked to antioxidant, anti‐inflammatory, anti‐senescent, and anti‐apoptotic effects in experimental models [21, 22, 23, 24]. Because these pathways were not directly evaluated in the present study, the observed ovarian protection cannot be attributed to a single molecular mechanism. In particular, the concurrent changes in TERT, FOXO3a, mTOR, and apoptosis‐related markers indicate treatment‐associated relationships but do not establish a causal telomerase–FOXO3a–mTOR signaling axis. Pharmacological or genetic pathway inhibition would be required to determine whether these molecular alterations are mechanistically linked. Beyond these pathways, cellular senescence has also been implicated as an important contributor to ovarian aging and age‐related deterioration of ovarian function [31].

The relevance of these findings to human ovarian physiology should be interpreted cautiously. In women, the primordial follicle pool is finite and cannot be regenerated to a clinically meaningful extent after substantial depletion. Chemotherapy‐induced loss of primordial follicles may therefore result in persistent reductions in ovarian reserve, premature ovarian insufficiency, infertility, and long‐term endocrine consequences. However, differences between rodents and humans in follicular dynamics, reproductive lifespan, ovarian reserve, and drug metabolism limit direct extrapolation of the present findings. Studies using human granulosa cells, ovarian cortical tissue, and ex vivo ovarian models are required to determine whether the treatment‐associated effects observed in rats are reproducible in human ovarian tissue.

The potential oncological implications of CAG administration should also be considered. Telomerase is active in many malignant cells and may contribute to tumor survival, chemotherapy resistance, and reduced apoptotic sensitivity [32, 33]. The present study was conducted in healthy rats and did not evaluate whether CAG altered the antineoplastic efficacy of CIS. Therefore, these findings cannot be directly translated into a fertility‐preservation strategy for patients receiving chemotherapy without confirmation in tumor‐bearing models and simultaneous assessment of tumor response.

The principal limitations of this study include the absence of direct ovarian telomerase activity and TERT mRNA measurements; lack of phosphorylated mTOR, AKT, and PI3K analyses; absence of pharmacological or genetic pathway inhibition; absence of complementary apoptosis assays, such as TUNEL staining, cleaved caspase‐3 analysis, or protein‐level validation by Western blot analysis; use of sampled consecutive sections rather than stereological follicle quantification; and lack of long‐term reproductive outcomes. The estrous‐cycle stage was also not incorporated into the interpretation of the ovarian endpoints. In addition, the separate results of the two vehicle‐control groups are provided in Table S1. Future studies incorporating direct ovarian telomerase assays, phosphorylated signaling proteins, pathway inhibition, complementary apoptosis analyses, functional fertility outcomes, and tumor‐bearing models are required to clarify the mechanism, reproductive efficacy, and oncological safety of CAG.

## 5 Conclusion

CIS‐induced ovarian toxicity was characterized by follicular depletion, structural injury, a shift toward pro‐apoptotic signaling, reduced ovarian TERT immunoreactivity, and alterations in the expression and subcellular distribution of FOXO3a and mTOR. CAG co‐treatment significantly attenuated the CIS‐induced changes in BAX, BCL‐2, and the BAX/BCL‐2 ratio, reduced nuclear phospho‐FOXO3a immunoreactivity, partially restored cytoplasmic total FOXO3a, and increased ovarian TERT immunoreactivity to a level comparable with that of the control group. In contrast, PCNA‐positive cell density, nuclear total FOXO3a, caspase‐3, total mTOR, and serum AMH showed only partial or non‐significant recovery.

These findings support a modulatory and partially protective effect of CAG against CIS‐induced ovarian injury. The observed increase in serum telomerase‐related ELISA measurements should not be interpreted as a direct measurement of telomerase activity. Overall, the findings indicate treatment‐associated changes in apoptotic balance, FOXO3a subcellular localization, mTOR immunoreactivity, and ovarian TERT immunoreactivity, but they do not establish a causal telomerase–FOXO3a–mTOR mechanism.

## Author Contributions

**Gökçe Nur Arık Erol:** conceptualization, investigation, methodology, data curation, writing – review and editing, writing – original draft. **Atiye Seda Yar:** conceptualization, methodology, data curation. **Gülnur Take Kaplanoğlu:** project administration, supervision.

## Conflicts of Interest

The authors declare no conflicts of interest.

## Supporting information


Supporting File

## Data Availability

The data that support the findings of this study are available from the corresponding author upon reasonable request.
