# Supplementary material for: Cycloastragenol Attenuates Cisplatin‐Induced Ovarian Toxicity in Rats: Associations With TERT Expression, Apoptotic Balance, and FOXO3a–mTOR‐Related Signaling
Source: J Biochem Mol Toxicol. 2026 Oct 25;40(11):e71152. doi: 10.1002/jbt.71152 (PMC13695996; doi:10.1002/jbt.71152)
Supplement: Supplementary file 1 [file JBT-40-e71152-s001.docx]

**Supplementary Table S1. Comparison of the Two Vehicle-Control Groups Before Pooling**

| **Outcome** | **Oral DMSO control (n=5) Median (Q1–Q3)** | **Intraperitoneal DMSO control (n=5) Median (Q1–Q3)** | **p value** |
| --- | --- | --- | --- |
| PCNA-positive cell density | 81.8 (79.4–89.0) | 74.4 (72.2–77.6) | 0.075 |
| Nuclear total FOXO3a H-score | 76.0 (72.0–80.0) | 78.0 (70.0–88.0) | 0.835 |
| Cytoplasmic total FOXO3a H-score | 80.0 (78.0–85.0) | 90.0 (88.0–91.0) | 0.173 |
| Nuclear p-FOXO3a (Ser253) H-score | 26.0 (21.0–28.0) | 25.0 (20.0–30.0) | 1.000 |
| Cytoplasmic p-FOXO3a (Ser253) H-score | 56.0 (48.0–60.0) | 50.0 (46.0–52.0) | 0.403 |
| Total mTOR H-score | 56.0 (54.0–58.0) | 60.0 (59.0–62.0) | 0.530 |
| Caspase-3 H-score | 30.0 (28.0–36.0) | 27.0 (22.0–31.0) | 0.403 |
| BAX H-score | 28.0 (26.0–30.0) | 30.0 (29.0–30.0) | 0.672 |
| BCL-2 H-score | 224.0 (210.0–230.0) | 132.0 (116.0–200.0) | 0.075 |
| BAX/BCL-2 ratio | 0.134 (0.122–0.152) | 0.190 (0.167–0.259) | 0.095 |
| TERT H-score | 58.0 (56.0–62.0) | 52.0 (49.0–58.0) | 0.401 |

Data are presented as median and interquartile range (Q1–Q3). The oral and intraperitoneal vehicle-control groups were compared using two-sided Mann–Whitney U tests. The animal was considered the experimental unit. For PCNA-positive cell density, the mean of five microscopic fields was calculated for each animal. No comparison reached statistical significance (p < 0.05).
